# Membrane order in the plasma membrane and endocytic recycling compartment

**DOI:** 10.1371/journal.pone.0188041

**Published:** 2017-11-10

**Authors:** David B. Iaea, Frederick R. Maxfield

**Affiliations:** 1 Department of Biochemistry, Weill Cornell Medical College, New York, NY, United States of America; 2 Weill Cornell Medical College, Rockefeller University, and Memorial Sloan Kettering Cancer Center Tri-Institutional Chemical Biology Program, New York, NY, United States of America; University of Nebraska Medical Center, UNITED STATES

## Abstract

The cholesterol content of membranes plays an important role in organizing membranes for signal transduction and protein trafficking as well as in modulating the biophysical properties of membranes. While the properties of model or isolated membranes have been extensively studied, there has been little evaluation of internal membranes in living cells. Here, we use a Nile Red based probe, NR12S, and ratiometric live cell imaging, to analyze the membrane order of the plasma membrane and endocytic recycling compartment. We find that after a brief incubation to allow endocytosis, NR12S is distributed between the plasma membrane and the endocytic recycling compartment. The NR12S reports that the endocytic recycling compartment is more highly ordered than the plasma membrane. We also find that the plasma membrane and the endocytic recycling compartment are differentially affected by altering cellular cholesterol levels. The membrane order of the plasma membrane, but not the endocytic recycling compartment, is altered significantly when cellular cholesterol content is increased or decreased by 20%. These results demonstrate that changes in cellular cholesterol differentially alter membrane order within different organelles.

## Introduction

The structure and function of cellular membranes is largely dictated by their lipid composition, including the amount of cholesterol [1]. Cholesterol is heterogeneously distributed among cellular organelles but is highly enriched in the plasma membrane (PM) and endocytic recycling compartment (ERC) [2]. In membranes, cholesterol interacts with the acyl chains and headgroups of surrounding lipids resulting in an increase in local membrane rigidity or order, and cholesterol depletion results in a decrease in local membrane order [3]. Cellular membranes are composed of hundreds of different lipids that vary in head group, acyl chain length and saturation level [4]. Additionally, lipids are non-randomly distributed in membranes and vary between the leaflets of the bilayer [5].

In recent years, there has been a growing interest in examining the membrane order of biological membranes in living cells [6]. Environmentally-sensitive probes, such as Laurdan [7], have been used to report local fluidity. However, due to the rapid redistribution of Laurdan among membranes, its application to studies of endosomal membranes has been limited [8].

**Fig 1:**
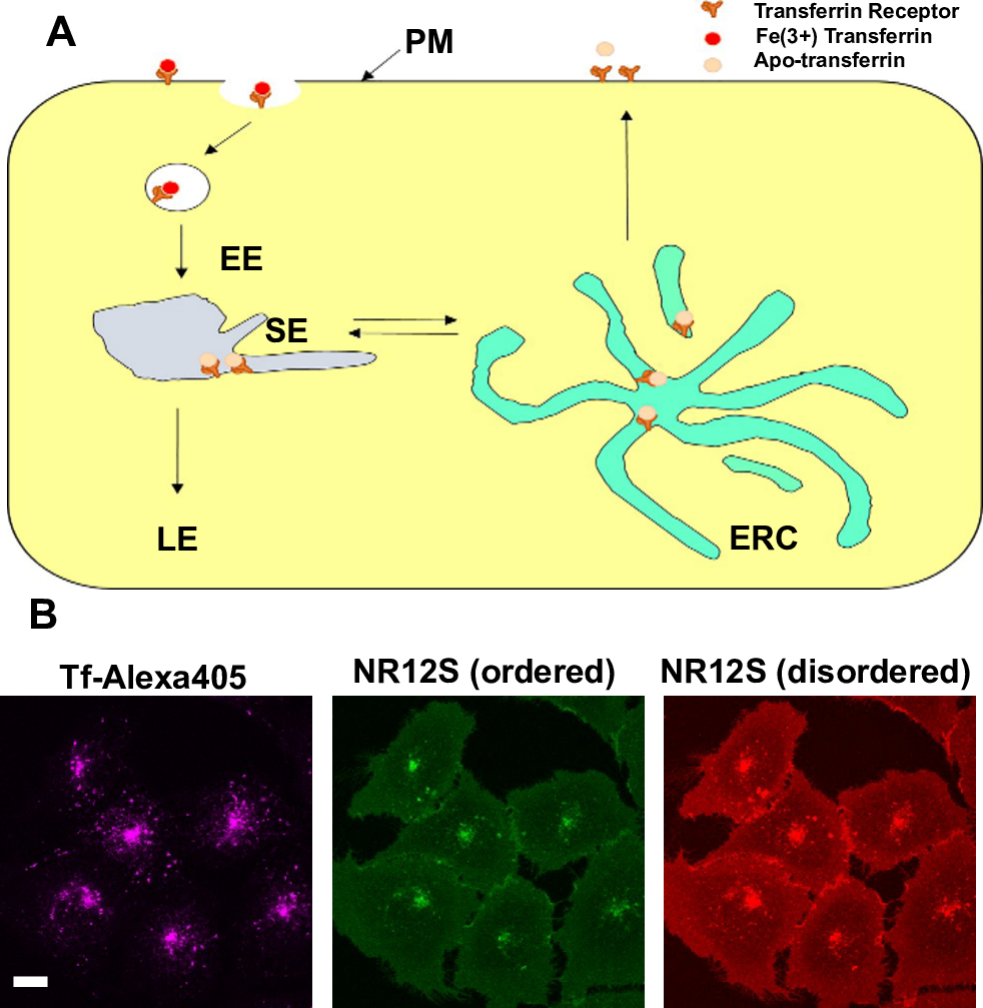
NR12S distributes between the PM and ERC. (A) Receptor-mediated endocytic pathway in non-polarized mammalian cells. Transferrin and its receptor are shown as an example. EE, early endosome; SE, sorting endosome; ERC, endocytic recycling compartment; LE, late endosome. (B) Representative maximum projection images of NR12S labeling in U2OS-SRA cells. The ERC is labeled with Alexa405-Tf. Scale bar is 10 μm.

Recently the Nile Red based probe, NR12S, has been developed and used to report the membrane order of the PM in living cells [9]. NR12S has a Nile Red-like moiety that is anchored in membranes by a long alkyl chain with a zwitterionic headgroup and does not flip across the bilayer [9]. NR12S allows for accurate measurements of membrane order [9], probably due to effects of water permeation on the spectroscopic properties of the fluorophore. In contrast to the PM, little is known about the biophysical properties of most intracellular membranes, including the ERC, which is part of the recycling itinerary for membrane proteins such as the transferrin receptor [10]. We have shown that fluorescent lipid analogs that, like NR12S, do not flip in the bilayer rapidly can be delivered to the ERC within a few minutes by nonselective endocytosis [11–13] (Fig 1A). In this study, we utilized the NR12S probe and ratiometric live cell imaging to monitor the membrane order of the PM and ERC, and we found that the ERC is more highly ordered than the PM. The membrane order of the PM appears to be altered to a greater extent than the ERC by changes in the cellular cholesterol content. These results indicate that cellular organelles differentially regulate their membrane order in response to alterations in cellular cholesterol levels.

## Materials and methods

### Materials

Alexa labeling kits were purchased from ThermoFisher. Human Transferrin (Tf) was purchased from Sigma. All tissue culture supplies were purchased from Invitrogen. All other chemicals were from Sigma. The medium used are as follows: Medium 2 (150mM NaCl, 5mM KCl, 1mM CaCl_2_, 1mM MgCl_2_ and 20mM HEPES, pH 7.4); M2glucose (Medium 2 containing 2mg/mL glucose).

### Cell culture

U2OS-SRA is a modified human osteosarcoma cell line that expresses the scavenger receptor A (SRA) [14]. U2OS-SRA cells were grown at 37°C in a 5% CO2 humidified incubator and in McCoys 5A medium supplemented with 10% fetal bovine serum, 1mg/mL geneticin as a selection for SRA, 100 units/mL penicillin, 100ug/mL streptomycin. Cells for confocal microscopy were plated on 35-mm plastic dishes, the bottoms of which were replaced with poly-D-lysine-coated coverslips. Cells were plated at 50–60% confluence for all experiments.

### Cholesterol modulation

U2OS-SRA cells were grown for 24 hours in cholesterol depletion medium (McCoys 5A medium similar to the growth medium but with 5% lipoprotein-deficient serum in place of fetal bovine serum, supplemented with 10 μM mevastatin). To overload cholesterol, U2OS-SRA cells were grown for 24 hours in metabolic overloading medium (McCoys 5A medium growth medium supplemented with 50 μg/mL acetylated-LDL and 30 μg Sandoz 58035).

### Free cholesterol measurement by GC/MS

Cellular lipids were extracted twice with hexane/2-propanol (3:2). During the first extraction, β-sitosterol was added as an internal standard for quantification. Dried lipids were resuspended in hexane and separated on a Varian Factor Four capillary column, using a Varian 400 GC/MS/MS system (14). The protein concentration after solubilization with 0.5 M NaOH was determined by the BCA protein assay.

### Fluorescence labeling

Human transferrin (Sigma) was iron-loaded and purified by Sephacryl S-300 (Pharmacia LKB) gel-filtration chromatography and conjugated to Alexa405 according to the manufacturer's instruction. To label cells with transferrin, cells were incubated with 20 μg/mL Alexa405–transferrin for 15 min at 37°C in M2glucose medium. NR12S was freshly prepared in M2glucose medium and then added to the cells at a final concentration of 0.3 μM. Cells were incubated for 7 min at 37°C in the dark. Following labeling, cells were washed and imaged in M2glcuose medium.

### Fluorescence microscopy

Cells were imaged on a Zeiss LSM 880, AxioObserver microscope equipped with a Plan-Apochromat 63× Oil 1.4 NA differential interference contrast (DIC) M27 objective in a humidified chamber at 37°C. Z-stacks were obtained using a step size of 0.31 μm. NR12S was excited using 514 nm laser and images corresponding to the green (520–580 nm) and red (585–650 nm) were recorded simultaneously using emission filters.

### NR12S image analysis

NR12S intensity in the plasma membrane and endocytic recycling compartment were measured in individual planes. Image planes were selected such that the plasma membrane or endocytic recycling compartment was the primary source of NR12S fluorescence. For the plasma membrane, peripheral regions of the cell were analyzed to exclude the endocytic recycling compartment fluorescence that was visible in the plasma membrane sections. All data were analyzed with MetaMorph image analysis software (Molecular Devices, Downingtown, PA).

## Results and discussion

### NR12S labels the PM and ERC

The fluorescence emission of NR12S is sensitive to the membrane environment. In more ordered membranes, NR12S fluorescence emission is blue shifted, while in disordered membranes the fluorescence emission spectra is red shifted (9). These alterations in the emission properties of NR12S allow for ratiometric green/red imaging to determine relative membrane order in cells. In this study, we modulated cholesterol levels using metabolic methods by growing human osteosarcoma cells (U2OS) stably expressing the scavenger receptor A (U20S-SRA cells) [14] in either lipoprotein deficient serum with the HMG-CoA reductase inhibitor mevastatin, or in medium supplemented with acetylated low density lipoprotein, which binds to SRA, and an acyl-CoA:cholesterol acyltransferase inhibitor, Sandoz 58–035 (see details in Materials and Methods). A previous study used cyclodextrin treatment to reduce cholesterol levels, but did not report the extent of cholesterol reduction (9).

To determine the cellular distribution of NR12S, we labeled the outer leaflet of the PM of living U2OS-SRA cells with 0.3 μM NR12S as described by Kucherak et al.[9]. After brief incubations, NR12S, like other fluorescent lipid analogs that do not flip spontaneously in the bilayer [11–13], is delivered to the ERC by endocytic processes, where it co-localizes with endocytosed transferrin (Fig 1). As the PM and ERC are highly enriched in cholesterol [2], we sought to analyze how the membrane order of these compartments changes following modulation of cellular cholesterol levels.

### Monitoring membrane order in living cells

**Fig 2:**
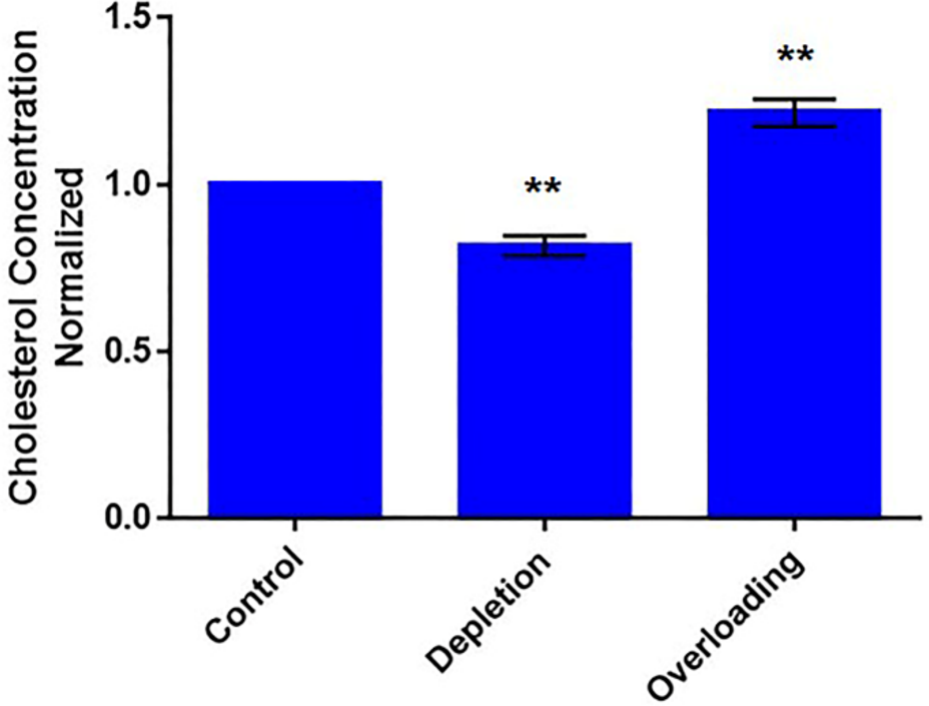
Metabolic modulation of cellular cholesterol levels. GC/MS measurement of free cholesterol levels in control, cholesterol depleted and overloaded U2OS-SRA cells. Cellular lipids were extracted and analyzed by GC/MS. Data represent averages (± SE) of three independent experiments normalized to control value. ** p < 0.01.

**Fig 3:**
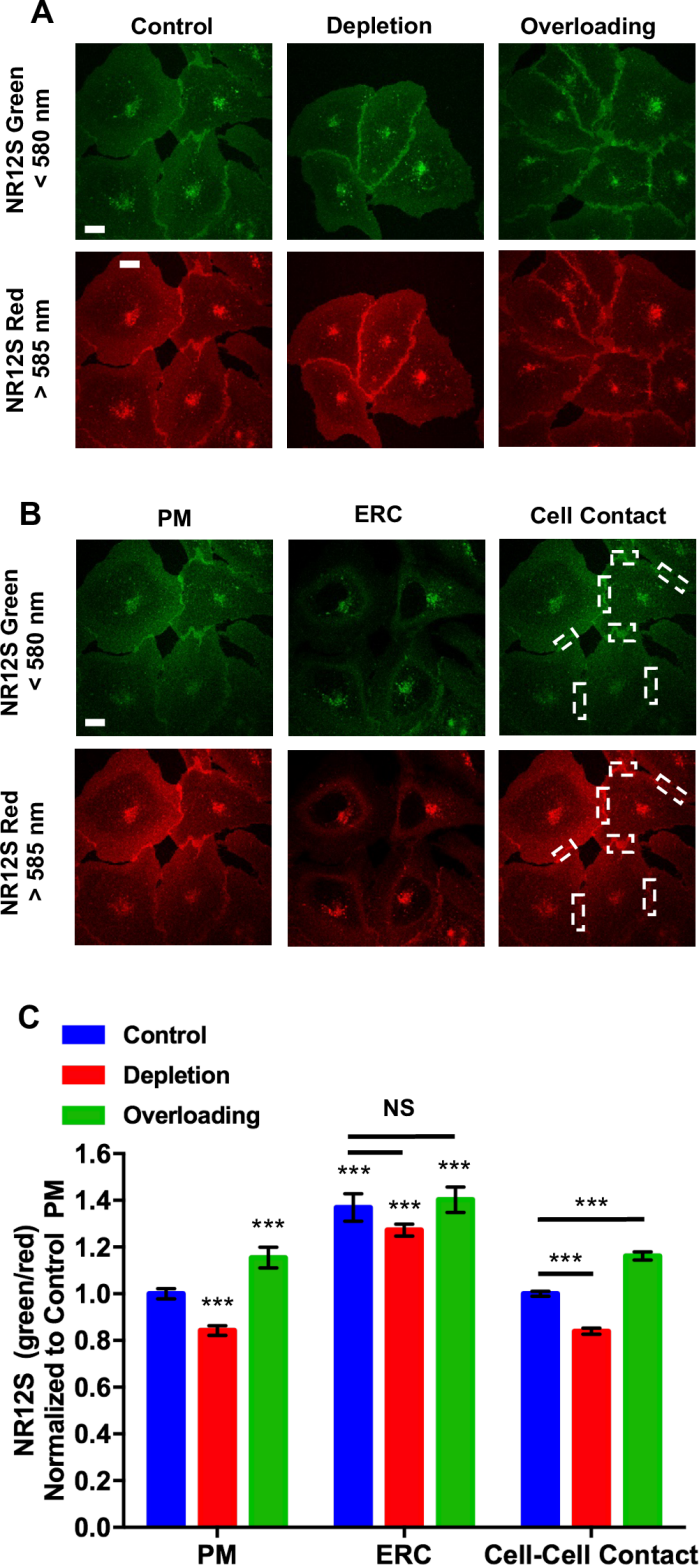
(A) Representative maximum projection images of NR12S labeling in control, cholesterol depleted, and cholesterol overloaded U2OS cells. Scale bar is 10 μm. (B) Representative planes used in the analysis of NR12S fluorescence intensity in the PM, ERC and Cell contact regions. Areas of cell contact points used in analysis are boxed. Scale bar is 10 μm. (C) Analysis of NR12S green/red ratio of the ERC and plasma membrane. NR12S green/red ratio was normalized to control PM. Data represent averages (± SE) of four independent experiments of at least 15 cells per experiment; NS, non-significant; *** p < 0.001 compared to control PM.

To modulate cellular cholesterol levels, we cultured U2OS-SRA cells under cholesterol depletion and cholesterol overloading conditions. Overnight cholesterol depletion resulted in a ~20% decrease in cellular cholesterol levels, while overloading increased cholesterol by ~20% (Fig 2). Using NR12S, we monitored changes in the membrane order of the PM and ERC following cholesterol modulation. Under all conditions, NR12S was distributed between the PM and ERC in U2OS-SRA cells (Fig 3A).

To analyze the green/red ratio of NR12S fluorescence in the PM and ERC, we collected a confocal stack of cells labeled with NR12S using a single excitation wavelength, and the emission for the green (ordered) and red (disordered) was collected simultaneously using emission filters (see details in Materials and Methods). The green/red ratio of NR12S for the PM and ERC as well as cell-cell contact points was determined using single planes where the fluorescence from the ERC, the PM, and PM at contact regions could be isolated from one another (Fig 3B). Because we are measuring fluorescence ratio values, variations in intensity due to the amount of dye do not affect the measurement. Comparing the NR12S green/red ratio of the ERC to the PM in control cells shows that the ERC has a green/red ratio about 40% higher than the PM (Fig 3C). This indicates that the ERC membrane is more ordered than the PM.

Analysis of the cholesterol depleted or overloaded U2OS-SRA cells shows that there was an alteration to the membrane order of the PM (Fig 3C). In the PM, NR12S green and red signal showed that following cholesterol depletion, NR12S PM emission was red shifted with a ~20% decrease in the NR12S green/red ratio, compared to control U2OS-SRA cells. This shift is consistent with a decrease in cellular cholesterol content and a reduction in membrane order [9]. Additionally, cholesterol overloading resulted in an increase in membrane order and a blue shift of NR12S. Consistent with these observations, measurements of cell-cell contact points from cholesterol depleted or overloaded cells demonstrated a similar shift in membrane order as the bulk plasma membrane measurement. We note that under our culture conditions (50–60% confluency), every cell we used for measurements was touching at least one other cells. We did not observe any dependence of the green/red ratio in the contact regions on the extent of cell-cell contact.

In the ERC, there was a small decrease in the green/red ratio when cholesterol was depleted (Fig 3C), but this difference did not reach statistical significance based on four experiments. The green/red ratio in the ERC was virtually unchanged upon cholesterol overloading. These data indicate that effects of alterations in cholesterol have a smaller effect on membrane order in the ERC than in the PM.

Cellular organelles are composed of complex mixtures of lipids that modulate their biophysical properties [1]. Unfortunately, the membrane composition of the ERC has not been well documented owing to difficulties of organelle purification. The abundance of cholesterol in the ERC [2] would be consistent with the membranes being highly ordered. Previous work has shown that GPI-anchored proteins are retained longer in the ERC compared to either the transferrin receptor or fluorescent lipid analogs [11], indicating that the ERC is highly ordered [15].

The retention of GPI-anchored proteins in the ERC was abolished when cellular cholesterol levels were reduced by ~ 50%. In the current study, when cholesterol levels are altered by ~20%, we observe that the membrane order of the PM, but not the ERC, is altered by modulation of cellular cholesterol levels. This indicates that the membrane properties of the PM are more influenced by changes in cholesterol than those of the ERC. Interestingly, it was shown recently that the effect of cholesterol on ordered lipid domain formation is dependent upon the phospholipid composition of the membranes [16, 17]. The retention of cholesterol and increased membrane order of the ERC implies that lipids may be differentially distributed between the PM and ERC. Enrichment of specific lipids, including cholesterol, in these organelles likely defines their unique biophysical properties.
